# Special Issue “Natural Plant Substances—Structural and Application Aspects: A Theme Issue in Honor of Professor Wieslaw Oleszek”

**DOI:** 10.3390/molecules27113430

**Published:** 2022-05-26

**Authors:** Jerzy Żuchowski, Anna Stochmal, Solomiia Kozachok, Andy J. Pérez, Łukasz Pecio

**Affiliations:** 1 Department of Biochemistry and Crop Quality, Institute of Soil Science and Plant Cultivation—State Research Institute, Czartoryskich 8, 24-100 Puławy, Poland; jzuchowski@iung.pulawy.pl (J.Ż.); asf@iung.pulawy.pl (A.S.); lpecio@iung.pulawy.pl (Ł.P.); 2 Departamento de Análisis Instrumental, Facultad de Farmacia, Universidad de Concepción, Concepción 4070386, Chile; aperezd@udec.cl

Dear Colleagues,

This Special Issue is dedicated to the 45th anniversary of Prof. Wiesław Oleszek’s scientific career, in recognition of his outstanding achievements in the field of natural plant metabolites, especially saponins.

Short Biography of Authors

Prof. Wiesław Oleszek was born in 1948 in Bełżyce, Poland. In 1975, he graduated from Maria Curie-Skłodowska University in Lublin, Poland, obtaining a Master’s degree in physics. In the same year, Prof. Oleszek started his work at the Institute of Soil Science and Plant Cultivation (IUNG), State Research Institute in Puławy, Poland. His scientific interests were related to the chemistry of plant metabolites. His doctoral thesis, titled “Alfalfa (*Medicago media* Pers.) and red clover (*Trifolium pratense* L.) root saponins and their allelopathic effect on wheat seedling growth” was prepared under the supervision of Prof. Marian Jurzysta, and defended in 1985 at IUNG. Prof. Oleszek, together with his supervisor, were one of the first Polish scientists specializing in isolation and determination of structures of saponins. During his work at the Institute, he took numerous foreign internships: New York State Agriculture Experiment Station, Cornell University, Food Science and Technology Department, USA (1980/1981; 1987); Institute of Food Research, Norwich, England (1989); INRA, Avignon, France (1993, post-doc position). Prof. Oleszek defended his habilitation thesis titled: “Saponins from the roots of alfalfa (*Medicago sativa* L.): chemistry, biological activity and determination” at IUNG, in 1991. In the years 1992–2010, he headed the Department of Biochemistry and Crop Quality at IUNG. Since 2010, Prof. Oleszek has been the Director of IUNG.

Prof. Oleszek was twice awarded by the Polish Academy of Sciences, obtaining a team award for research on chemotaxonomy in Trigonellae in 1989, and an award for research on plant saponins in 2001. In 1995, he was awarded the Foundation for Polish Science prize—Nutris 95. Prof. Oleszek and his research team participated in many national projects, e.g., LCAgri, Operative Programme for Development of Eastern Poland, and international projects, e.g., FATEALLCHEM in 5th Framework Program; SAFEWASTE, NUTRASNACK and FEEDSEG in 6th Framework Program; PROFICIENCY (coordinator), OSCAR, EMAP and FOODSEG in 7th Framework Program; he was also a coordinator of two H2020 projects: BIO ECON and BIOEASTsUP.

Prof. Oleszek was a supervisor of eight PhD students at the Department of Biochemistry and Crop Quality at IUNG, as well as a reviewer of dozens of PhD and habilitation theses. A reviewer of numerous national and international scientific papers and projects, Prof. Oleszek has also worked in evaluation teams of the State Committee for Scientific Research and the National Science Centre (Poland), and the European Science Foundation.

Prof. Oleszek is an active participant of the European Research Communities. In 1995, he was one of the founding members of the International Allelopathic Society based in Cadiz, Spain. Prof. Oleszek was the first representative of the Central and Eastern Europe in the Phytochemical Society of Europe, based in Cambridge in 1994–1998, the General Secretary of this Society in 2002–2007, vice President of the European Phytochemical Society in 2008–2010, and its President in 2010–2012. Prof. Oleszek was also a member of the editorial board of several journals: “Allelopathy Journal”, “Phytochemistry Reviews”, “Phytochemistry Letters”, “Phytochemical Analysis”, “Open Journal of Biochemistry, Frontiers in Sustainable Food Systems (Crop Biology and Sustainability)”, and the “Polish Journal of Food and Nutrition Sciences”.

Prof. Oleszek is listed as one of the top 2% most cited scientists in the world according to a Stanford University ranking (2020).

This Special Issue comprises of 17 publications. Seven articles are focused on chemical profiling of extracts or extract fractions from different medicinal and crop plants, as well as diverse aspects of their biological activity. Kozachok et al. [1] performed LC-MS analyses of extracts from three species of rupturewort (*Herniaria* L.), medicinal plants known for their triterpenoid saponins, and compared levels of 38 non-saponin compounds, mostly flavonoids and phenolic acid derivatives. Antioxidant and anti-inflammatory activities of the extracts were also determined. Another team investigated phenolics and some more hydrophobic compounds (LC-MS and GC-MS) in extracts from different organs of wild onions, *Allium galanthum* and *A. turkestanicum*, as well as their antimicrobial, antioxidant, and anti-tyrosinase activities [2]. Masullo et al. characterized the chemical profile of extracts from the South American fruit *Pouteria lucuma* (LC-MS), as well as isolated the detected phenolic compounds and determined their antioxidant activity [3]. Similarly, Ślusarczyk et al. performed LC-MS analyses of extracts from *Coleus amboinicus*, a spice and medicinal plant from the tropical zone, and determined their antioxidant properties [4]. Chemical profiles (LC-MS) and antioxidant and anticancer activity of extracts from sweet and hot peppers (*Capsicum annuum*) were determined by Chilczuk et al. [5]. The publication of Żurek et al. describes the identification and quantification of phenolic compounds (LC-MS) in extracts from the fruit, leaves and flowers of six species of hawthorn (*Crataegus* L.). The anticancer activity of the extracts was also investigated [6]. The last article in this group describes identification (LC-MS) and quantitation of saponins, flavonoids, and proanthocyanidins in leaves and flowers of *Hedysarum coronarium*, a legume fodder crop [7].

The remaining publications cover a broad range of topics. Pharmacology-related issues are continued in four articles. Anticancer, antimicrobial, and antioxidant activity of fractions of extracts from sea buckthorn (*Elaeagnus rhamnoides*; syn. *Hippophae rhamnoides*) leaves and twigs, as well as their composition, were characterized by Stochmal et al. [8]. Grabowska et al. isolated two new tritepenoid saponins, glycosides of serjanic acid from *Chenopodium hybridum*, and tested their anticancer properties on numerous cell lines [9]. Another article describes isolation of an isoquinoline alkaloid palmatine from roots of *Berberis cretica* and determination of its anticancer properties against human breast cancer cell lines [10]. Antioxidant and anti-inflammatory activities of a melatonin-containing plant preparation and the synthetic melatonin were compared by Kukuła-Koch et al. [11]. The study of Horbowicz et al. compares the effects of natural senescence and treatment with methyl jasmonate on levels of flavonoids, phenolic acids, and terpene trilactones in leaves of *Ginkgo biloba* [12]. In the second article associated with plant physiology research, Dashbaldan et al. investigated changes in the distribution and composition of triterpenoids and steroids in developing *Rosa rugosa* hips [13]. Najar et al. investigated the crop yield and essential oil composition of two organically grown chemotypes of thyme (*Thymus vulgaris*), in a three-year experiment [14]. The publication of Wrona et al. concerned the optimisation of conditions of the supercritical CO_2_ extraction of bioactive compounds from alfalfa [15]. One article is completely different from the remaining, as it does not describe plant natural products. Koch et al. determined levels of several macro- and microelements in samples of black teas from different countries and locations, and evaluated the possibility of application of chemometric methods to distinguish the origin of black teas on the basis of their mineral composition [16].

Finally, this Special Issue includes a review on the structure and bioactivity of *Yucca* saponins, and analytical methods used for determination of these compounds [17].

From the scope of the research presented in the articles, it is clear that the research on plant specific substances, of which Prof. Oleszek is an undoubted pioneer and animator, is still an important object of interest for many contemporary scientists. We also observe a dynamic growth of interest in research at the interface of phytochemistry and medicine.
